# Isoharringtonine Induces Apoptosis of Non-Small Cell Lung Cancer Cells in Tumorspheroids via the Intrinsic Pathway

**DOI:** 10.3390/biom10111521

**Published:** 2020-11-06

**Authors:** Ji Hae Lee, So-Young Park, Wonbin Hwang, Jee Young Sung, Myoung-Lae Cho, Jaegal Shim, Yong-Nyun Kim, Kyungsil Yoon

**Affiliations:** 1 Division of Translational Research, National Cancer Center, Goyang 10408, Korea; jhlee5@ncc.re.kr (J.H.L.); 75360@ncc.re.kr (S.-Y.P.); hwb25@naver.com (W.H.); sungjy@ncc.re.kr (J.Y.S.); jaegal@ncc.re.kr (J.S.); ynk@ncc.re.kr (Y.-N.K.); 2 College of Pharmacy, Graduate School of Pharmaceutical Sciences, Ewha Womans University, Seoul 03760, Korea; 3 National Institute for Korean Medicine Development, Gyeongsan 38540, Korea; meanglae@nikom.or.kr

**Keywords:** isoharringtonine, NR4A1, apoptosis, tumorspheroids, NSCLC

## Abstract

Lung cancer is the major cause of cancer-associated death worldwide, and development of new therapeutic drugs is needed to improve treatment outcomes. Three-dimensional (3D) tumorspheroids offer many advantages over conventional two-dimensional cell cultures due to the similarities to in vivo tumors. We found that isoharringtonine, a natural product purified from *Cephalotaxus koreana* Nakai, significantly inhibited the growth of tumorspheroids with NCI-H460 cells in a dose-dependent manner and induced apoptotic cell death in our 3D cell culture system. On the other hand, A549 tumorspheroids displayed low sensitivity to isoharringtonine-induced apoptosis. Nuclear receptor subfamily 4 group A member 1 (NR4A1) is an orphan nuclear receptor known to regulate proliferation and apoptosis of cancer cells. We observed that knockdown of NR4A1 dramatically increased isoharringtonine-induced cancer cell death in A549 tumorspheroids by activating the intrinsic apoptosis pathway. Furthermore, treatment with combined isoharringtonine and iNR4A1 significantly inhibited multivulva formation in a *Caenorhabditis elegans* model and tumor development in a xenograft mouse model. Taken together, our data suggest that isoharringtonine is a potential natural product for treatment of non-small cell lung cancers, and inhibition of NR4A1 sensitizes cancer cells to anti-cancer treatment.

## 1. Introduction

Lung cancer is one of the most frequently diagnosed types of cancer and is the leading cause of cancer-associated death worldwide [1]. Non-small cell lung cancers (NSCLCs) account for approximately 85% of human lung cancer [2] and have a five-year survival rate of only 15.9% [3]. Most NSCLC patients are diagnosed in an advanced stage due to difficulties with early detection, and the common treatment options include cytotoxic chemotherapy, target therapy, and immunotherapy [2,4,5]. Despite recent advancements of target therapy and immunotherapy, the population of applicable or responsive patients is small, and resistance to treatment develops rapidly [2,4]. Thus, new effective therapeutic drugs or combination strategies are needed.

Traditional herbal medicine is a good source of therapeutic agents and has long been used in clinics, with general acceptance of efficacy and safety. We screened a natural product library consisting of an array of single compounds extracted from traditional herbal medicinal materials for growth inhibition of NSCLC tumorspheroids. Isoharringtonine (IHT), one of the most effective natural products, is an alkaloid extracted from the leaves of *Cephalotaxus koreana* Nakai. *Cephalotaxus* has long been used in traditional medicine, as the fruits and leaves are effective against parasitic infection and insect bites, respectively [6]. In China, *Cephalotaxus* has been applied as treatment of human malignant tumors [7,8]. Alkaloids extracted from *Cephalotaxus harringtonia* have been reported to have anti-tumor activity against murine leukemia cells [9,10]; among them, harringtonine, IHT, and homoharringtonine were shown to inhibit protein synthesis [11,12,13]. Homoharringtonine induced apoptosis in human leukemia cells [14,15,16], and Omacetaxin, a semi-synthetic formulation of homoharringtonine were Food and Drug Administration (FDA)-approved for treatment of chronic myeloid leukemia with resistance and/or intolerance to two or more tyrosine kinase inhibitors in 2012 [17]. Homoharringtonine and IHT have been reported to inhibit transcription factor, signal transducer, and transcription 3 (Stat3) activation in gefitinib-resistant NSCLC and breast cancer cells, respectively [18,19].

Transcription factors play a critical role in tumorigenesis, tumor progression, and drug response. Therefore, it is considered a good anti-cancer strategy to target oncogenic transcription factors [20]. Nuclear receptor subfamily 4 group A member 1 (NR4A1, Nur77, Tr3, NGF1-B) is a product of immediate-early gene and orphan nuclear receptor associated with various cellular processes such as cell proliferation, apoptosis, inflammation, metabolism, and vascular remodeling [21,22,23,24]. Regulation of apoptosis by NR4A1 appears to be complicated. Nuclear export of NR4A1 elicits non-genomic pro-apoptotic function in cancer cells by direct interaction with B-cell lymphoma 2 (Bcl-2) and exposure of Bcl-2 Homolog 3 domain to initiate the intrinsic apoptosis pathway [25,26]. Nuclear NR4A1 receptors have a growth-inhibitory effect by inducing pro-apoptotic and anti-proliferative genes [27,28]. On the contrary, NR4A1 exerts its anti-apoptotic function by increasing the expression of Survivin and Bcl-2 at the transcription level [29]. The role of NR4A1 in regulating apoptosis appears to be tissue- or tumor type-specific, and more research is needed to elucidate this complex regulation.

Anti-cancer drug development is a challenge, illustrated by less than 5% approval rates of new cancer drugs. This limited success is due to the inability of an in vitro system to reproduce the complexity and heterogeneity of human solid tumors [30,31]. Three-dimensional (3D) tumorspheroids offer advantages in resembling in vivo solid tumors including cell to cell and cell to extracellular matrix interactions, cell polarity, and hypoxia [30,32,33]. Tumorspheroids also possess several in vivo features such as diffusion gradients of drugs, oxygen, and nutrients, all of which can influence chemotherapeutic efficacy [30,32,33]. We employed a 3D in vitro tumorspheroid model to screen the natural product library for anti-tumor activity to better predict the biology and drug responses of human solid tumor in vivo.

Here we report that IHT extracted from the leaves of *Cephalotaxus koreana* Nakai effectively reduces the growth of NSCLC tumorspheroids and increases apoptotic cell death via the intrinsic pathway. Furthermore, NR4A1 knockdown extensively induces apoptosis in tumorspheroids that display low sensitivity to isoharringtonine-induced cancer cell death.

## 2. Materials and Methods

### 2.1. Preparation of IHT

**Scheme 1:**
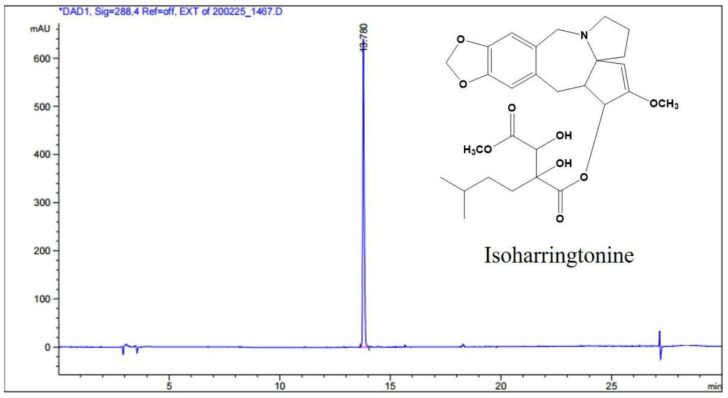
High performance liquid chromatography (HPLC) chromatogram and chemical structure of isoharringtonine from *Cephalotaxus koreana* Nakai.

The dried leaves of *Cephalotaxus koreana* Nakai (1.5 kg) were extracted two times with methanol (MeOH) for 3 h at room temperature (2 × 7000 mL). The crude MeOH extract (228.0 g) was suspended in distilled water (3000 mL) and then suspension was partitioned with the same volume of n-hexane (Hx), ethyl acetate (EtOAc), and butanol (BuOH). The BuOH soluble fraction (67.0 g) was separated into 10 fractions (CKB 1–10) by silica gel column chromatography (Kieselgel 60, 70–230 mesh, Merck, Darmstadt, Germany) with a gradient of chloroform (CHCl_3_) and MeOH (20:1 to 0:1). The CKB 4 fraction was re-chromatographed using silica gel with CHCl_3_ and MeOH (10:1) to yield 15 fractions (CKB 4-1–4-15). The subfraction CKB 4-11 was purified by reversed phase-high performance liquid chromatography (RP-HPLC, J’sphere ODS H80, 20 × 250 mm, 4 μm, YMC, Tokyo, Japan) with acetonitrile (ACN) and 0.03M ammonium carbonate (50:50) to obtain active compound IHT (71.0 mg, purity: 99%). The structure of IHT was determined with comparison of those spectroscopic data in the previous literature [34]. The NMR spectra were recorded on a JEOL ECX-500 spectrometer, operating at 500 MHz for 1H and 125 MHz for ^13^C NMR spectrum (JEOL Ltd., Tokyo, Japan). The determination of HPLC system was equipped with Agilent 1260 series (Agilent Inc., Palo Alto, CA, USA) with a quaternary pump, a degasser, an injector, a column thermostat, a diode array detector (DAD), and an evaporative light scattering detector (ELSD). The gradient elution was carried out with 0.1% (*v*/*v*) TFA in water (solvent A) and 0.1% (*v*/*v*) TFA in acetonitrile (solvent B). The gradient conditions for the samples were analyzed from 95% A/5% B at 3 min to 0% A/100% B at 30 min. The flow rate and injection volume was 0.5 mL/min and 3 μL, respectively. HPLC chromatogram is shown with the structure of IHC in Scheme 1.

### 2.2. Characterization Data for IHT

White powder; ESI-MS 532 [M+H]+, molecular formula C_28_H_37_NO_9_; ^1^H-NMR (500 MHz, CD3OD) δ 6.62 (1H, s, H-17), 6.58 (1H, s, H-14), 5.97 (1H, d, *J* = 9.7 Hz, H-3), 5.82 (1H, d, *J* = 1.1 Hz, H-18a), 5.72 (1H, d, *J* = 1.1 Hz, H-18b), 5.16 (1H, s, H-16), 3.85 (1H, d, *J* = 9.8 Hz, H-4), 3.24 (1H, 1H, m), 3.19 (1H, m, H-8a), 2.91 (1H, m, H-11a), 2.84 (1H, td, *J* = 11.7, 7.2 Hz, H-10a), 2.60 (2H, m, H-8b, H-10b), 2.39 (1H, dd, *J* = 14.4, 6.9 Hz, H-11b), 1.98 (1H, m, H-7a), 1.90 (1H, m, H-6a), 1.78 (1H, m, H-6b), 1.40 (2H, m, H-1″b, H-3″), 1.20 (1H, m, H-2″a), 0.98 (1H, m, H-2″b), 0.82 (1H, d, *J* = 6.7 Hz, H-4″), 0.81 (1H, d, *J* = 6.7 Hz, H-5″); ^13^C NMR (125 MHz, CD3OD) 173.5 (C-1′), 172.9 (C-4′), 160.1 (C-2), 148.1 (C-15), 147.1 (C-16), 134.3 (C-13), 129.9 (C-12), 114.2 (C-14), 110.9 (C-17), 102.0 (C-18), 100.6 (C-1), 80.7 (C-2′), 75.9 (C-3), 75.3 (C-3′), 72.2 (C-5), 57.7 (C-19), 56.2 (C-4), 54.5 (C-8), 52.2 (C-5′), 49.8 (C-10), 43.7 (C-6), 34.8 (C-1″), 32.7 (C-2″), 32.0 (C-11), 29.4 (C-3″), 23.2 (C-4″), 22.7 (C-5″), 20.7 (C-7).

### 2.3. Cell Lines and Cell Culture

A549, NCI-H460, Calu-6, NCI-H1299, and NIC-H596 cells were obtained from the American Type Culture Collection (Rockville, MD, USA) and cultured in Roswell Park Memorial Institute 1640 (RPMI1640, HyClone, South Logan, UT, USA) supplemented with 10% fetal bovine serum (HyClone, South Logan, UT, USA) and 1× penicillin-streptomycin (Gibco, Waltham, MA, USA). Cells were maintained at 37 °C in a humidified atmosphere of 5% CO_2_. To generate tumorspheroids, A549 and NCI-H460 cells were seeded at the density of 7 × 10^3^ or 1.5 × 10^3^ per well into 96-well round-bottom ultra-low attachment plates (ULA, Corning, NY, USA), respectively, and centrifuged at 1000 rpm for 10 min. Spheroids were cultured in RPMI culture medium supplemented with Matrigel (0.5%, BD Biosciences, San Jose, CA, USA) to promote compact spheroid formation. When the spheroid size reached 350–450 μm in diameter, day 2 or day 3 after spheroid formation, spheroids were treated with IHT.

### 2.4. siRNA Transfection and IHT Treatment

Cells were transfected with 20 ηM of siRNA using Lipofectamine 2000 (Invitrogen, Waltham, MA, USA) following the manufacturer’s protocol. The sequences of the NR4A1 siRNA are as follows: siNR4A1 #1, 5′-GAGCUAUUCCAUGCCUACG-3′; siNR4A1 #2, 5′-GGAUACUGGAUACACCCGU-3′. siRNA for negative control (siNC) was purchased from Bioneer (Seoul, Korea). To generate NR4A1 knockdown tumorspheroids, cells were replated in ultra-low attachment (ULA) plate after siRNA transfection for 6 h. Two days later, tumorspheroids were treated with IHT.

### 2.5. Cell Viability Assay

For 2D cell viability assay, cells were plated at 50% confluence in 96-well white/clear flat bottom microplate and treated with the indicated concentrations of IHT. After 48 h incubation, CellTiter-Glo reagent (Promega, Madison, WI, USA) was directly added into each well. For 3D cell viability assay, spheroids were removed from the ULA plate and placed two spheroids per well of a 96-well white microplate, after 48 and 72 h treatment with IHT. CellTiter-Glo 3D reagent (Promega, Madison, WI, USA) was added into each well and incubated at room temperature for 25 min. The luminescence signal was measured by a luminometer (VICTOR X, PerkinElmer, Waltham, MA, USA).

### 2.6. Live/Dead Cell Staining

Spheroids were stained by the LIVE/DEAD™ Viability/Cytotoxicity Kit (Thermo Scientific, Rockford, IL, USA) following the manufacturer’s instructions. Images were obtained by an Operetta High Content Screening System (PerkinElmer, Waltham, MA, USA) and analysis was performed using Harmony 3.5.2 software (PerkinElmer, Waltham, MA, USA).

### 2.7. Quantitative Real-Time PCR

Total RNA was extracted using the RNeasy Mini Kit (Qiagen, Hilden, Germany), cDNA was synthesized using cDNA synthesis kit (Dyne Bio, Seoul, South Korea) and FastStart essential DNA Green Master mix (Roche, Basel, Switzerland) were used for the PCR reaction. Primer sequences used for PCR were as follows: NR4A1 (F: 5′-AGGGCTGCAAGGGCTTCT-3′ and R: 5′-GGCAGATGTACTTGGCGTTTTT-3′); GAPDH (F: 5′-TGATGACATCAAGGTGGTGAAG-3′ and R: 5′-TCCTTGGAGGCCATGTGGGCCAT-3′). PCR reactions were performed as follows: 95 °C denaturation for 5 min, followed by 40 cycles at 94 °C for 10 s, 60 °C for 10 s, 72 °C for 10 s, followed by a 9 min extension at 72 °C.

### 2.8. Apoptosis Analysis by Enzyme-Linked Immunospecific Assay (ELISA)

Cell Death Detection ELISAPLUS Kit (Roche, Basel, Switzerland) was used to detect apoptosis according to the manufacturer protocol. This kit quantifies cytoplasmic histone-complexed DNA fragments (mono- and oligonucleosomes) after induced apoptosis. Briefly, spheroids were treated with IHT for 72 h. To obtain the cytoplasmic fractions, single spheroid was lysed in 200 μL lysis buffer. The lysate of A549 cells was used after 4-fold dilution. Absorbance was measured at 405 ηm (reference wavelength at 490 ηm).

### 2.9. Apoptosis Analysis by Flow Cytometry with Annexin V/7-Amino-Actinomycin D (7-AAD) Staining

For the apoptosis assay, dissociated cells were washed with phosphate buffered saline (PBS, HyClone, South Logan, UT, USA) and resuspended in annexin-binding buffer at 1 × 10^5^ cells/100 μL. Thereafter, cell suspension was incubated for 15 min with 5 μL of Phycoerythrin (PE)-Annexin V and 5 μL of 7-AAD solution (BD Pharmingen, San Diego, CA, USA) at room temperature. After incubation, cells were analyzed by flow cytometry. The Data were analyzed with Cell Quest Software (BD bioscience, San Jose, CA, USA).

### 2.10. Flow Cytometer Analysis for Mitochondria Potential

Spheroids were dissociated into a single cell using 0.25% trypsin- ethylenediaminetetraacetic acid (EDTA) (Gibco, Waltham, MA, USA) at 37 °C for 5 min and suspended in PBS. For the measurement of mitochondrial membrane potential changes, cells were incubated with 20 ηM tetraethylbenzimidazolylcarbocyanine iodide (JC-1) in PBS for 15–30 min at 37 °C, followed by flow cytometer analysis (FACSCalibur; Becton Dickinson Bioscience, NJ, USA).

### 2.11. Immunofluorescence Staining

Cells were seeded in 24-well culture plates and treated with IHT or transforming growth factor-β (TGF-β) (R&D Systems, Minneapolis, MN, USA) for 5 h. After washing with PBS, cells were fixed with 4% paraformaldehyde (Biosesang, Seongnam, South Korea) for 15 min. Cells were permeabilized with 0.1% Triton X-100 (Sigma-Aldrich, St. Louis, MO, USA,) for 15 min, and blocked with 10% goat serum (Vector Laboratories, Inc., Burlingame, CA, USA) for 15 min. Primary and secondary antibodies were prepared in 1% BSA in PBS and incubated for overnight or 2 h. The primary antibody used was anti-NR4A1 (1:100, #3960, Cell signaling Technology, Beverly, MA, USA), and secondary antibody used was Alexa Fluor^®^ 488 goat anti-rabbit IgG (1:50, A11008, Invitrogen, MA, USA). Pictures were taken under a LSM780 (Carl Zeiss, Oberkochen, Germany) confocal microscope.

### 2.12. Western Blot Analysis

Whole cell lysates were prepared in radio immunoprecipitation assay (RIPA) buffer, supplemented with protease inhibitor cocktail, phosphatase inhibitor (Calbiochem, San Diego, CA, USA), phenylmethylsulfonyl fluoride (PMSF, Sigma-Aldrich, St. Louis, MO, USA), and dithiothreitol (DTT, Sigma-Aldrich, St. Louis, MO, USA). Protein concentrations were determined using a micro bicinchoninic acid (BCA) protein assay kit (Thermo Scientific, Rockford, IL, USA). Equal amounts of protein were separated by 8–12% sodium dodecyl sulphate-polyacrylamide gel electrophoresis (SDS-PAGE) and transferred to a polyvinylidene difluoride (PVDF) membrane (BioRad, Hercules, CA, USA) using a wet transfer device (BioRad, Hercules, CA, USA). Antibodies for the following proteins were used in this study: β-Actin (sc-477778), p53 (sc-126), Bax (sc-526) (Santacruz Biotechnology, Dallas, TX, USA); cleaved caspase-7 (Asp198) (#9491), cleaved poly (ADP-ribose) polymerase (PARP) (#9541), X chromosome-linked inhibitor of apoptosis (XIAP) (#2045), Survivin (#2808), p21 (#2947), phospho-Akt (pAkt) (#9275), cleaved caspases-9 (#9501), caspases-9 (#9502), caspases-8 (#9746) and pStat3 (#9145), (all obtained from Cell Signaling Technology, Beverly, MA, USA). The secondary antibodies used were horseradish peroxidase (HRP)-conjugated goat anti-rabbit immunoglobulin G (IgG) (sc-2004, Santacruz Biotechnology, Dallas, TX, USA) and goat anti-mouse IgG (SA001-500, GenDEPOT, Barker, TX, USA). Bands were developed using enhanced chemiluminescent method with HRP substrate (Thermo Scientific, Rockford, IL, USA).

### 2.13. RNA Interference and Drug Treatment in Caenorhabditis Elegans (C. Elegans)

The *jgIs25* strain was cultured in nematode growth medium (NGM) plates at 20 °C. The *nhr-6* cDNA was amplified by RT-PCR using C48D5.1-01 (5′ TATCTGCAGATGGAACAACTTAGTATTCAAACGG 3′) and C48D5.1-02 (5′ ATATCTAGATTAAAAGGCAGCCGGGCCC 3′) primers, and wild-type total RNA was used as a template. The *nhr-6* RNAi plasmid was constructed in L4440 vector from the amplified *nhr-6* PCR product using restriction enzyme sites, *PstI* and *XbaI*. The empty L4440 vector was used as a control for RNAi experiments. Dose-specific IHT treatment was performed in 96 well plates with a final volume of 100 μL. 0.5% DMSO was used as a control. Synchronized L1 larvae were harvested, washed 3 times with M9 buffer and aliquoted into each well. After OP50 and cholesterol were added, they were incubated at 20 °C for 3 days. Grown adults were transferred to new NGM plates and Muv-bearing animals were counted after 24 h recovery. For the simultaneous treatment of isoharringtonine and *nhr-6* RNAi, *nhr-6* RNAi plates were prepared and 5 μM IHT or 0.1% DMSO was added. After transferring four L4 larvae, Muv-bearing animals were counted from mature F1 progeny. All experiments were performed three times, and the *p* value compared to the control was calculated by *t-test*.

### 2.14. Mouse Xenograft Study

A549 (5 × 10^6^) cells were suspended in 100 μL PBS and mixed with 50 μL Matrigel. The mixtures were subcutaneously inoculated into 6-week-old BALB/c nude mice (Orient Bio, Seongnam, Korea). When the tumor size reached 100 to 150 mm^3^, the mice were divided randomly into four treatment groups. siRNA (50 ρmole) was injected into the xenograft tumor via electroporation using NEPA21 Super Electroporator (Nepa gene Co., Chiba, Japan) once per week for 4 weeks; and IHT (20 μg/kg) was intratumorally injected into xenograft tumor twice weekly for 4 weeks. The injection volume of siRNA and IHT was 20 μL. The tumor size was monitored twice a week up to 6 weeks. Tumor volume was calculated with the following formula: V (mm^3^) = length × width × width/2. This study was reviewed and approved by the Institutional Animal Care and Use Committee (IACUC) of National Cancer Center Research Institute (NCC-19-505, 6/11/2019; NCC-20-505B, 20/8/2020). National Cancer Center Research Institute is an Association for Assessment and Accreditation of Laboratory Animal Care International (AAALAC International) accredited facility and abide by the Institute of Laboratory Animal Resources (ILAR) guide.

### 2.15. Statistical Analysis

Data are presented as mean ± standard deviation (SD) or standard error of the mean (SEM) of three independent experiments. Comparison between two groups was performed using Student’s *t*-test and significance was defined as a *p* value < 0.05. For survival analysis, Kaplan–Meier Plotter [35] was used to investigate the association of NR4A1 mRNA expression and overall survival in lung cancer patients.

## 3. Results

### 3.1. IHT Inhibits the Growth of NSCLC Tumorspheroids

**Figure 1:**
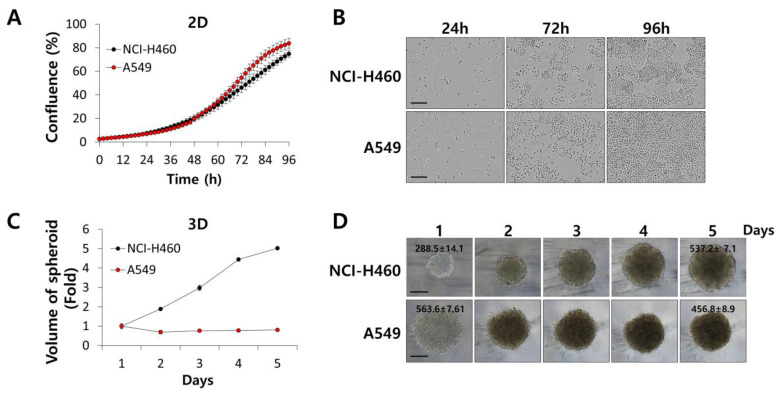
Cell growth was different in two-dimensional (2D) monolayer culture and three-dimensional (3D) tumorspheroids. (**A**,**B**) A549 and NCI-H460 cells were plated at a density of 2 × 10^4^ cells per well of 96-well plates. Cell confluence was measured every 2 h for 96 h using the IncuCyte live cell analysis system. Representative images of cells were produced at 24, 72, and 96 h. (**C**,**D**) A549 and NCI-H460 cells were seeded in 96-well ultra-low attachment (ULA) plates and measured by diameter of spheroids at the indicated times. Spheroid volume was calculated as 4/3 × π × r^3^. All data are presented as mean ± SD. Spheroid morphology was examined by light microscopy. Representative number is the diameter (μm) of spheroids. Scale bars, 200 μm.

First, we examined the growth properties of A549 and NCI-H460 tumorspheroids in comparison to those of A549 and NCI-H460 cells grown in a 2D culture. As shown in Figure 1, A549 and NCI-H460 cells grew exponentially in the 2D system, with similar proliferation rates (Figure 1A,B). In a 3D culture system, NCI-H460 tumorspheroids grew fast from a diameter of 288.5 ± 14.1 μm to that of 537.2 ± 7.1 μm over 4 days (Figure 1C,D). On the contrary, A549 tumorspheroids did not grow throughout the culture period (Figure 1C,D). Zaconi et al. reported that homogeneous volume and spherical shape of tumorspheroids were important to produce consistent data [33]. We maintained tumorspheroids in a spherical shape smaller than 550 μm in diameter, as seen in Figure 1D, and conducted a luminescent cell viability assay to measure the growth-inhibitory activity of IHT in NSCLC tumorspheroids.

**Figure 2:**
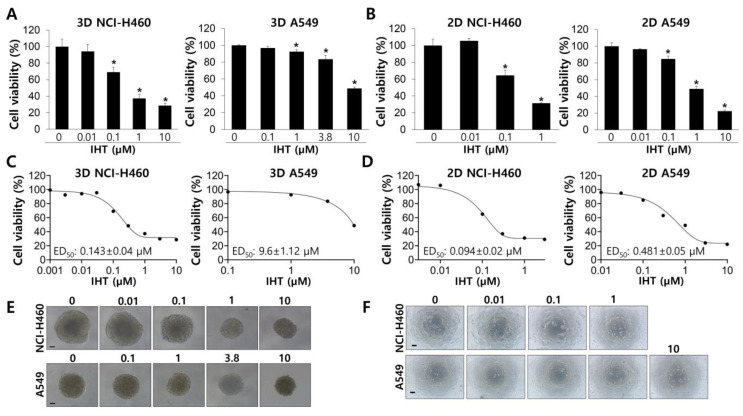
Isoharringtonine (IHT) inhibits the growth of non-small cell lung cancer (NSCLC) cells in 2D and 3D culture systems. (**A**) A549 and NCI-H460 cells were seeded in ULA plates and grown for 2 days before IHT treatment with the indicated concentrations. After 72 h, cell viability was measured by the CellTiter-Glo 3D cell viability assay kit. *, significantly different from control (*p* < 0.05). (**B**) 2D-cultured cells were exposed to IHT at the indicated concentrations for 48 h, and cell viability was examined by the CellTiter-Glo assay. *, significantly different from control (*p* < 0.05). (**C**,**D**) The median effective dose (ED_50_) values of IHT were obtained from dose-response curves generated by GraphPad Prism. (**E**,**F**) Representative images were collected before viability assay. Scale bars, 100 μm. All data are presented as mean ± SD.

IHT effectively inhibited growth of NCI-H460 tumorspheroids at a concentration as low as 0.1 μM in a dose-dependent manner, with a median effective dose (ED_50_) of 0.143 ± 0.04 µM (Figure 2A,C,E). A549 tumorspheroids were much more resistant to IHT-induced inhibition than were those of NCI-H460, with an estimated ED_50_ of 9.6 ± 1.12 µM (Figure 2A,C,E). IHT inhibited viability of NSCLC cells, especially A549 cells, more effectively in 2D culture than in 3D culture (Figure 2B,D,F), consistent with previous reports in various cancer cell types [36,37,38,39]. This might be due to lack of cellular contacts and high rates of cell proliferation in the 2D system (Figure 1), resulting in ED_50_ values of IHT of 0.094 ± 0.02 µM and 0.481 ± 0.05 µM in NCI-H460 and A549 cells, respectively (Figure 2D). These results demonstrate that the characteristics of cellular proliferation and responses to compounds are different between 2D and 3D culture conditions.

### 3.2. IHT Induces Apoptotic Cell Death in NCI-H460 Tumorspheroids and iNR4A1 Sensitizes A549 Tumorspheroids to IHT-Induced Apoptosis

**Figure 3:**
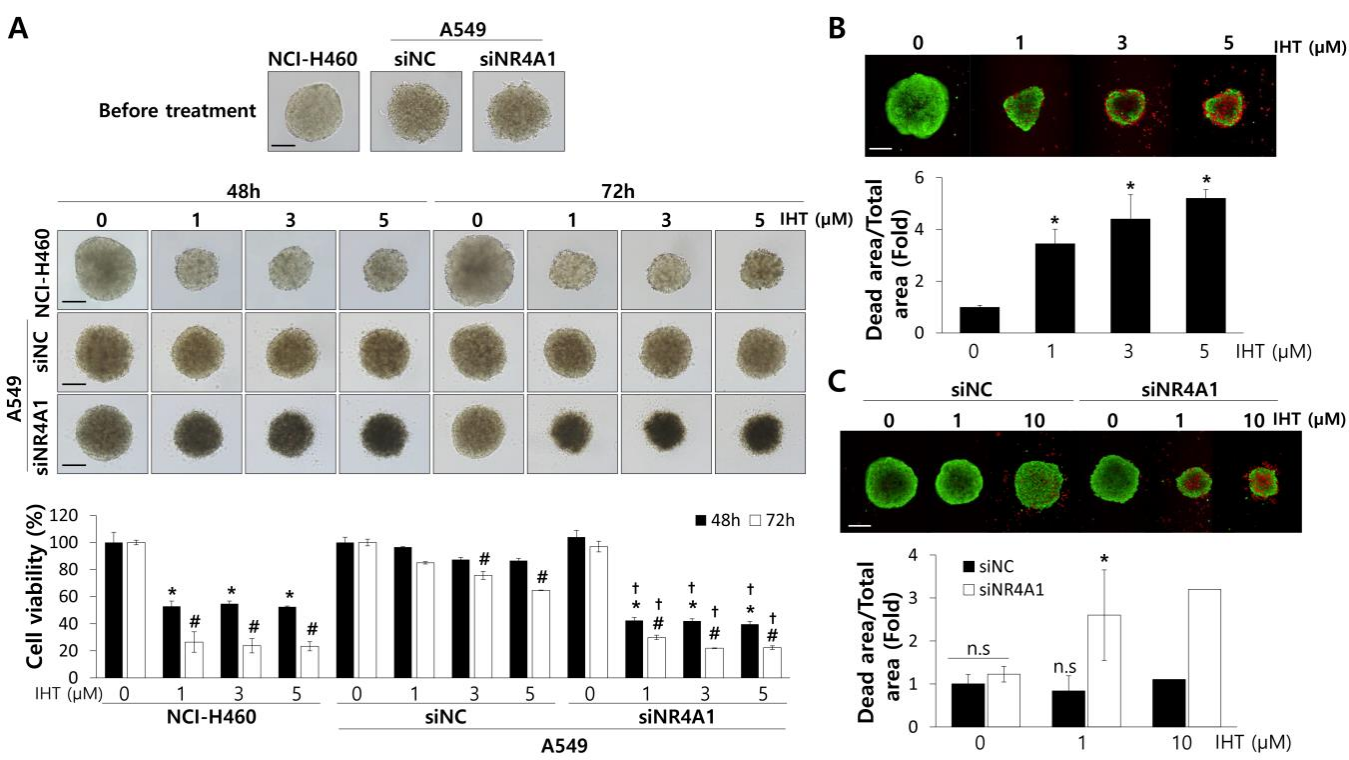
Treatment of IHT in NCI-H460 and with combination of IHT and nuclear receptor subfamily 4 group A member 1 (NR4A1) knockdown in A549 effectively repressed the growth of tumorspheroids and enhanced cell death. (**A**) Two days after ULA plating, NCI-H460 and siRNA-transfected A549 tumorspheroids were treated for 48 and 72 h with the indicated concentrations of IHT. Cell viability was measured by the CellTiter-Glo 3D cell viability assay. Scale bars, 200 μm. *, significantly different from control at 48 h (*p* < 0.05); #, significantly different from control at 72 h (*p* < 0.01); †, significantly different from siRNA for negative control (siNC) (*p* < 0.01). NCI-H460 (**B**) and A549 (**C**) tumorspheroids were treated with IHT for 72 h. Live cells were stained green with calcein-acetoxymethyl ester (AM), while dead cells were stained red with ethidium homodimer-1 (EthD-1). Cell images were collected at a magnification of 100× using an Operetta High Content Screening (HCS) System. Scale bar: 200 μm. *, significantly different from control (*p* < 0.05). All data are presented as mean ± SD.

Dysregulated gene expression is a hallmark of cancers and is created mainly by transcriptional regulators including transcription factors. NR4A1 regulates proliferation and apoptosis in cancer cells by transcriptionally regulating target genes [40]. It recently has been reported that NR4A1 is overexpressed in tumors from NSCLC patients and is associated with tumor recurrence [41], suggesting its involvement in chemotherapy resistance. We silenced NR4A1 expression in A549 tumorspheroids (Supplementary Figure S3), which displayed modest inhibition by IHT. In addition, treatment with IHT dramatically inhibited growth of A549 tumorspheroids and resulted in extensive cell death (Figure 3A,C). NCI-H460 cells in tumorspheroids also experienced extensive cell death, as expected (Figure 3B), but further responses upon siNR4A1 treatment were not observed (Supplementary Figure S1).

**Figure 4:**
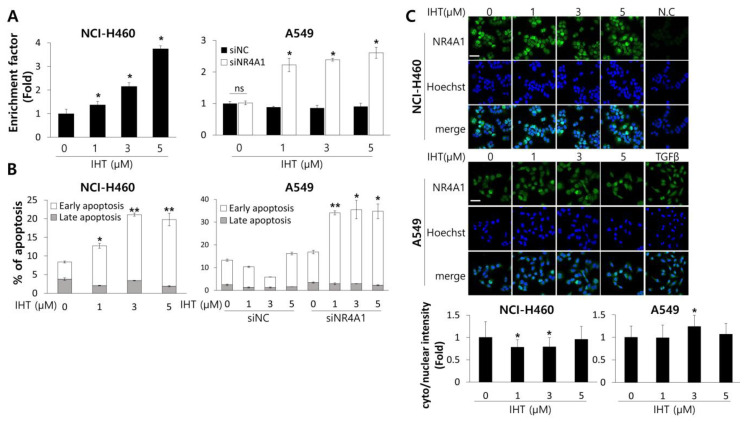
Treatment with IHT in NCI-H460 and with a combination of IHT and NR4A1 knockdown in A549 tumorspheroids increased apoptotic cell death. (**A**) Two days after ULA plating, NCI-H460 and siRNA-transfected A549 tumorspheroids were treated with the indicated concentrations of IHT for 72 h, and apoptosis was analyzed using the Cell Death detection ELISA kit. *, significantly different from control (*p* < 0.05). n.s = not significant. (**B**) Dissociated tumorspheroids at 72 h after IHT treatment were analyzed by flow cytometry. Apoptotic cells are presented as a percentage of Annexin V-positive/7-amino-actinomycin D (7-AAD)-negative cells (early apoptosis) and of Annexin V/7-AAD double-positive cells (late apoptosis). *, significantly different from control (*p* < 0.05). **, significantly different from control (*p* < 0.01). (**C**) 2D-cultured A549 and NCI-H460 cells were treated with IHT for 5 h. Expression and localization of NR4A1 were assessed by immunofluorescence staining. The secondary antibody used for NR4A1 was labeled by Alexa 488 (green), and nuclei were stained by Hoechst 33342 (blue). Scale bars, 50 μm. *, significantly different from control (*p* < 0.05). All data are presented as mean ± SD.

To confirm whether the IHT-induced cell death was ascribed to apoptosis, we quantified cytoplasmic histone-complexed DNA fragments and found that NCI-H460 tumorspheroids treated with IHT displayed increased apoptosis (Figure 4A). In A549 tumorspheroids, only combination treatment of IHT and siNR4A1 induced apoptotic cell death and did so at an as low as 1 μM concentration (Figure 4A). We also performed flow cytometric analysis of cells stained with Annexin V and 7-AAD. IHT increased the population of NCI-H460 cells dissociated from tumorspheroids in the early stage of apoptosis (Figure 4B). Treatment with IHT alone failed to increase the apoptotic population of A549 tumorspheroids, whereas additional NR4A1-knockdown drove apoptotic cell death (Figure 4B). NR4A1 is a nuclear transcription factor but is exported outside the nucleus to exert pro-apoptotic function [25,26]. Immunofluorescence staining for NR4A1 in NCI-H460 and A549 cells treated with various doses of IHT revealed that NR4A1 remained mainly in the nucleus, though 3 μM of IHT-treated cells showed a slight increase in cytoplasmic NR4A1. The TGF-β-treated group was included as a positive control for nuclear export of NR4A1 (Figure 4C).

### 3.3. IHT Induces Apoptosis of NSCLC Tumorspheroids via the Intrinsic Pathway

**Figure 5:**
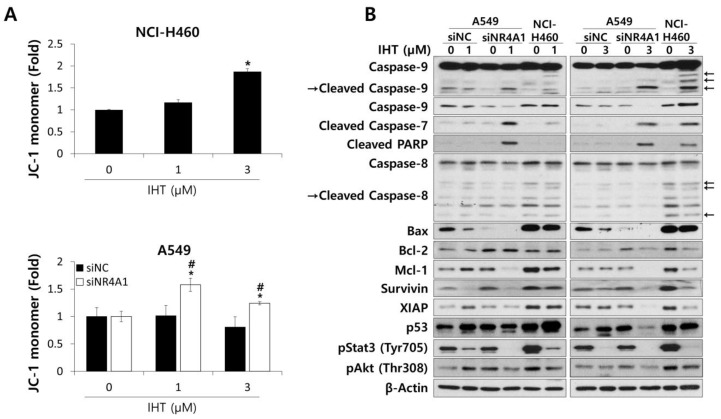
IHT induced mitochondria-mediated apoptosis in 3D-cultured NCI-H460 cells and A549 cells treated with siNR4A1. (**A**) To detect alteration of mitochondria membrane potential, tumorspheroids were dissociated into single cells after IHT treatment for 48 h. Cells were stained with tetraethylbenzimidazolylcarbocyanine iodide (JC-1) and analyzed by flow cytometry. *, significantly different from siNR4A1 control (*p* < 0.05); #, significantly different from siNC control (*p* < 0.01). All data are presented as mean ± SD. (**B**) Whole cell lysates were prepared from NCI-H460 and siRNA-transfected A549 tumorspheroids 48 h after IHT treatment. The levels of apoptosis-related proteins were analyzed by western blotting. β-Actin was used as a loading control.

To analyze the apoptotic pathway, we first determined if the treatment with IHT affected mitochondrial integrity. As shown in Figure 5A, the population of cells displaying monomeric JC-1 in NCI-H460 tumorspheroids treated with IHT was significantly higher than that of untreated controls, indicating that IHT induced the mitochondrial depolarization of NCI-H460 cells and thus activated the intrinsic pathway of apoptosis [42]. Treatment of A549 tumorspheroids with IHT did not change the mitochondrial potential except at the highest concentration, while the combination of iNR4A1 and IHT increased the percentage of cells with monomeric JC-1 (Figure 5A).

Consistent with this observation in IHT-treated NCI-H460 tumorspheroids, cleavage of caspase-9 was significantly increased at the 3 μM concentration and to a much lesser extent at the 1 μM concentration (Figure 5B). Cleavage of the downstream effectors caspase-7 and PARP also was detected, though no significant changes were observed in caspase-8, which is activated in the extrinsic apoptotic pathway (Figure 5B). These data confirm that IHT induces apoptosis via the intrinsic pathway in NCI-H460 tumorspheroids. Combined NR4A1 knockdown in NCI-H460 tumorspheroids did not affect any changes induced by IHT treatment at the apoptosis-related protein level (Supplementary Figure S2), consistent with the viability data (Supplementary Figure S1). In A549 tumorspheroids, treatment with IHT alone did not induce cleavage of caspase-9, caspase-7, or PARP. However, combination with siNR4A1 remarkably induced cleavage of these effectors, even at the 1 μM concentration (Figure 5B).

Proteins responsible for inducing apoptosis via the intrinsic pathway were analyzed by western blotting to uncover the mechanisms related to IHT-induced apoptosis. Bcl-2 family members including anti-apoptotic and pro-apoptotic proteins are responsible for the mitochondrial apoptotic pathway by opening mitochondrial permeability transition pores. When a mitochondrial membrane is damaged, cytochrome c is released, resulting in activation of caspase-9 and, in turn, the effector caspase-3/7, which ultimately results in apoptosis [43]. In NCI-H460 tumorspheroids treated with IHT, pro-apoptotic Bax appeared to be reduced slightly, whereas anti-apoptotic Bcl-2 and myeloid cell leukemia 1 (Mcl-1) distinctively decreased (Figure 5B).

Apoptotic signals also are modulated by the inhibitors of apoptosis (IAPs) family. Expression of Survivin and XIAP was reduced upon 1 μM IHT treatment of NCI-H460 tumorspheroids, and further decrease was observed with 3 μM IHT (Figure 5B). Survivin binds caspase-9 and blocks apoptosis, and XIAP inhibits both caspase-9 and caspase-3/7 [44]. Taken together, the findings suggest that IHT induces the intrinsic apoptotic pathway by reducing the expression of Bcl-2, Mcl-1, XIAP, and Survivin at the protein level in NCI-H460 tumorspheroids.

It has been reported that p53 repressed Mcl-1, Bcl-2, and Survivin at the transcriptional level [45]; thus, we analyzed the changes of p53 level and found that IHT treatment increased the protein level of p53 in NCI-H460 tumorspheroids (Figure 5B).

On the other hand, in A549 tumorspheroids treated with IHT, only Survivin was downregulated (Figure 5B), indicating that a decrease in the level of Survivin upon IHT treatment may not be enough to activate apoptosis in A549 tumorspheroids. Interestingly, the combination of iNR4A1 with IHT decreased the expression of Mcl-1 and XIAP as in NCI-H460 tumorspheroids treated with IHT (Figure 5B). The p53 level decreased with the combination treatment (Figure 5B), suggesting that p53 is not associated with apoptosis in A549 cells, and that the basal levels of p53, Bax, and Bcl-2 were lower in A549 than in NCI-H460.

IHT has not been studied extensively in human cancers, but a recent study reported that it inhibited the phosphorylation of Stat3 and proliferation of breast cancer cells [19]. Consistently, the present study showed that IHT dramatically inhibited the phosphorylation of Stat3 in both IHT-treated NCI-H460 and A549 tumorspheroids (Figure 5B), though this does not explain the differences in apoptotic phenomena between these two NSCLC tumorspheroids. It has been reported that Akt in various NSCLC cells is constitutively active and promotes cell survival [46]. Treatment of NCI-H460 tumorspheroids with 3 μM IHT resulted in a decrease in the level of phospho-Akt. In A549 tumorspheroids, phospho-Akt level was decreased by the combination of iNR4A1 and 3 μM IHT, indicating that active Akt plays a role in cancer cell survival of NSCLC tumorspheroids.

Altogether, our data demonstrated that IHT decreased the expression of Bcl-2, Mcl-1, Survivin, and XIAP and increased p53 to activate the intrinsic apoptosis pathway in NCI-H460 tumorspheroids. In addition, decrease in expression of Mcl-1 and XIAP was the key event for apoptosis via the intrinsic pathway in A549 tumorspheroids treated with IHT and iNR4A1.

### 3.4. IHT with iNR4A1 has Anti-Tumor Effects In Vivo and Expression of NR4A1 in Lung Cancer Tissues Correlates with Patient Survival

**Figure 6:**
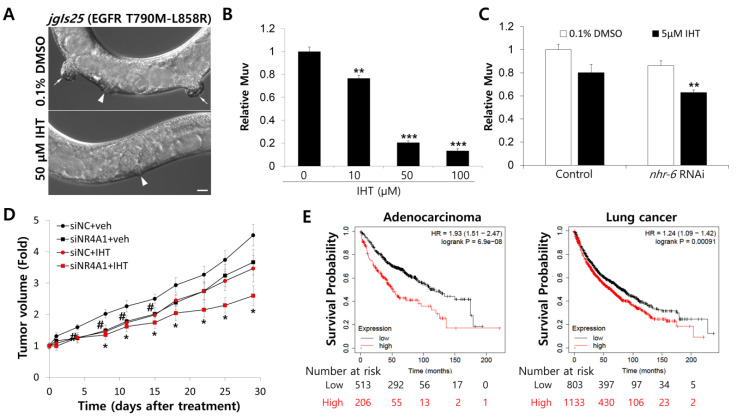
Combination of NR4A1 knockdown and IHT treatment synergistically inhibited multivulva (Muv) formation and mouse xenograft tumor growth. (**A**) The adult animal model of *jgIs25* treated with DMSO or IHT was observed using a differential interference contrast (DIC) microscope. Arrowhead = normal vulva. Arrow = hyperplasia. (**B**) The synchronized L1 larvae of *jgIs25* (epidermal growth factor receptor (EGFR) T790M-L858R) were treated with IHT at the indicated concentration in liquid culture. Worms with Muv were counted at adult stage, and 0.5% DMSO was used as a control. **, significantly different from control (*p* < 0.01); ***, significantly different from control (*p* < 0.001). Data are presented as mean ± SEM. (**C**) IHT treatment was combined with *nhr-6* RNAi. Four L4 larvae of *jgIs25* were transferred to DMSO, *nhr-6* RNAi, IHT, or *nhr-6* RNAi + IHT plates, and mature F1 progeny were counted. For this, 0.1% DMSO and 5 μM IHT were used. **, significantly different from control (*p* < 0.01); ***, significantly different from control (*p* < 0.001). Data are presented as mean ± SEM. (**D**) A549 cells (5 × 10^6^) were implanted subcutaneously into BALB/c nude mice. When the tumor size reached 100 to 150 mm^3^, siRNAs were delivered into the tumors via electroporation once a week, and IHT was injected into the tumors twice a week for 4 weeks. Tumors were measured at the indicated time, and tumor volume was calculated as described in Materials and Methods. #, significantly different between siNC + vehicle and siNC + IHT (*p* < 0.05); *, significantly different between siNC + vehicle and siNR4A1 + IHT (*p* < 0.05). Data are presented as mean ± SEM. (**E**) The association between NR4A1 mRNA expression and overall survival of adenocarcinoma and lung cancer patients was analyzed using Kaplan–Meier plot. HR: hazard ratio.

To investigate the anti-cancer effect of IHT in vivo, we employed a *C. elegans* model. Human NSCLC carrying epidermal growth factor receptor (EGFR) mutations in the kinase domain including L858R are highly responsive to EGFR tyrosine kinase inhibitors (TKIs) [47]. Treatment of human NSCLC with EGFR mutations including L858R with EGFR-TKIs has proved very effective [47]. However, resistance develops fast, and one of the mechanisms for this is acquisition of a second mutation, T790M [47]. The *C. elegans jgIs25* strain expresses human EGFR with two mutations of T790M and L858R and displays the multivulva (Muv) phenotype, which is an indicator of hyperplasia [48]. Treatment with IHT inhibited the Muv formation of *jgIs25* without adverse effects on animal growth at concentrations of 10 μM or more (Figure 6A,B). Next, to investigate the relationship between the anti-cancer effect of IHT and iNR4A1 in a nematode model, the *C. elegans* NR4A1 homologue NHR-6 was knocked down by RNAi. Treatment of *jgIs25* worms with IHT at a concentration of 5 μM did not inhibit Muv formation; however, with knockdown of NHR-6, an ortholog of human NR4A1, IHT significantly reduced Muv formation (Figure 6A). These results suggest that IHT is a promising anti-cancer drug to overcome EGFR-TKI resistance, and that NR4A1 plays a role in increasing resistance to IHT.

We tested the anti-tumor activity of IHT with iNR4A1 in a xenograft mouse model. Tumors were induced by subcutaneous injection of A549 cells into the dorsal area of BALB/c nude mice to reach 100–150 mm^3^ in volume, treated with IHT and siNR4A1 intratumorally, and monitored for 4 weeks. NR4A1 knockdown in tumors was confirmed by measuring mRNA level (Supplementary Figure S3). As shown in Figure 6D, the combined treatment significantly suppressed tumor growth compared to controls, but IHT or siNR4A1 alone did not. These data confirm that combination treatment of IHT and iNR4A1 inhibits NSCLC growth in vivo.

An association between clinical outcomes of lung cancer patients and NR4A1 expression was examined using the Kaplan–Meier plot [35]. This revealed that the level of NR4A1 mRNA was correlated inversely with the overall survival of patients with adenocarcinoma or any type of lung cancer (Figure 6E). Combined with our data, these observations suggest that NR4A1 is a candidate molecule for targeting NSCLC.

## 4. Discussion

According to Global Cancer Statistics 2018, lung cancer ranks number one in cancer incidence, comprising 11.6% of the total cases and 18.4% of total cancer death worldwide [1]. NSCLC includes adenocarcinoma, squamous cell carcinoma, large cell carcinoma, and other small subsets of lung cancer classified by immunohistochemistry for guiding treatment following World Health Organization (WHO) classification. In an advanced stage of NSCLC, cancer tissues will be analyzed for targetable mutations such as EGFR and anaplastic lymphoma kinase (ALK) to apply an appropriate TKI. If a targetable mutation is not found and the expression of programmed cell death ligand 1 (PD-L1) is positive, immunotherapy will be employed. For patients with low PD-L1 expression, standard chemotherapy is the choice. Cytotoxic chemotherapy is used for the rest of patients and is administered as a second-line treatment or as an adjuvant for surgery or radiation [2,49,50,51]. Despite these advancements of NSCLC treatment, innate or required resistance is difficult to overcome. Thus, new mechanism-based anti-cancer drugs or combination strategies are needed.

Signaling molecules and transcription factors are frequently mutated or dysregulated in cancer, mediating aberrant gene expression. Currently inhibitors of cell surface receptors or upstream kinases such as EGFR and BRAF are used as target therapy in various types of cancer and accomplish remarkable improvement in cancer treatment. However, resistance emerges through various mechanisms including bypass signaling or secondary mutations [20,52,53]. Transcription factors function at the merging endpoint of multiple signaling pathways and are difficult to bypass. In addition, they regulate multiple downstream targets playing a role in cancer development and progression [20,52,53]. For these reasons, targeting transcription factor drivers in cancer will be a good strategy for new cancer treatment, alone or in combination with other cancer therapies.

NR4A1 is reported to have both oncogenic and tumor suppressor functions in human cancer. These opposing effects of NR4A1 depend on the type and stage of cancer. For example, low NR4A1 expression in aggressive lymphoma was associated with poor overall survival, and enforced expression of NR4A1 induced apoptosis and suppressed xenograft tumor growth [54]. In addition, NR4A1 protein expression is decreased in triple-negative breast cancer (TNBC), and overexpression of NR4A1 inhibited TNBC growth and invasiveness [55]. On the contrary, it is reported that NR4A1 is overexpressed in bladder, breast, pancreatic, colon, and lung cancer [24,29,56,57,58] and is associated with poor survival in breast and lung cancer patients [41,59]. Zhu et al. reported that the protein level of NR4A1 was upregulated in NSCLC tissues compared with matched normal tissues, and elevated NR4A1 expression was correlated with clinical stage and tumor recurrence [41]. They also analyzed the correlation of NR4A1 protein level and survival of NSCLC patients and showed that overall survival and progression-free survival were shorter in NSCLC patients with high NR4A1 expression [41]. These observations and our data suggest that NR4A1 is a potential target for NSCLC treatment.

In our 3D culture system, dissimilar growth properties were displayed in the tumorspheroids of A549 and NCI-H460 NSCLC cell lines, resembling slow-growing and fast-growing tumors, respectively (Figure 1). At a low concentration, IHT inhibited the proliferation of NCI-H460 effectively and induced p21, a cell cycle inhibitor (Supplementary Figure S4), indicating that IHT induced inhibition of cell growth and apoptotic cell death depending on dose applied in fast growing cells. To determine the growth characteristic is a key determinant of IHT sensitivity, we compared NSCLC tumorspheroids with various growth rates. IHT significantly inhibited cell viability in growing tumorspheroids at low concentrations, 0.1 and 0.3 µM, not in non-growing tumorspheroids (Supplementary Figure S5), probably resulting from the inhibition of cell proliferation. At a relatively high concentration, 1 µM, IHT effectively inhibited cell viability in non-growing NCI-H596 tumorspheroids, and to the contrary, at the highest concentration, 10 µM, cell viability of non-growing tumorspheroids were repressed more than that of growing tumorspheroids. In addition, NCI-H460 tumorspheroids grew slower than Calu-6 but IHT inhibited cell viability of NCI-H460 much more efficiently (Figure 2D and Supplementary Figure S5). These observations suggest that in addition to the property of tumorspheroid growth, the efficacy of IHT is influenced by other cell type-specific factors in 3D culture, which need to be determined.

It was reported that drug-resistant genes were expressed at higher levels in A549 cells than in NCI-H460 [60]. Integrins protect cells from apoptosis by suppressing the p53 activation [61]. Recently, it has been reported that integrin β4 reduces chemosensitivity by inhibiting cisplatin-induced p53 activation in 3D cultures of HCT116 [62]. This effect was not observed in a 2D culture system. Our RNA sequencing data showed the basal expression of integrin β4 greater than 5-fold higher in A549 tumorspheroids than in NCI-H460 (data not shown). In addition, in our results, higher expression of pro-apoptotic p53 and downstream Bax was confirmed in NCI-H460 tumorspheroids than in A549 (Figure 5B). We speculated that A549 tumorspheroids were more resistant to IHT than NCI-H460, possibly due to p53 inhibition by integrin β4.

Apoptosis is an important process to maintain normal cellular homeostasis, and dysregulation of apoptosis results in diseases such as cancer or degenerative disorders. Apoptosis is executed through the intrinsic mitochondrial pathway activating caspase-9 and the extrinsic death receptor pathway activating caspase-8. Bid cleavage by caspase-8 is thought to link these extrinsic and intrinsic pathways to release cytochrome c from the mitochondria [63,64,65]. The Bcl-2 family of proteins comprises critical modulators in the intrinsic apoptosis pathway by regulating release of cytochrome c, ultimately resulting in cleavage of caspase-3 and -7. IAPs inhibit activation of those effector caspases and are involved in chemoresistance [64,65]. Many anti-apoptotic proteins in the intrinsic apoptosis pathway, such as Bcl-2, B cell lymphoma-extra large (Bcl-xL), Mcl-1, and some IAP proteins, are overexpressed in human cancers. Inhibitors of those molecules currently are in clinical trials [64,65]. IHT is an attractive candidate to develop as an anti-cancer drug due to its decrease in expression of Bcl-2, Mcl-1, XIAP, and Survivin in NCI-H460 tumorspheroids and its downregulation of Mcl-1 and Survivin in A549 with siNR4A1 to activate the intrinsic apoptosis pathway (Figure 5).

## 5. Conclusions

In this study, we demonstrated the anti-tumor activity of IHT in in vitro 3D tumorspheroids of NSCLC and in in vivo *C. elegans* and xenograft mouse models. IHT activated apoptotic cell death in NSCLC tumorspheroids via the intrinsic mitochondrial pathway, especially by decreasing expression of anti-apoptotic proteins in Bcl-2 and IAP families. In NSCLC tumorspheroids, with low sensitivity to IHT, combination with NR4A1 knockdown dramatically increased IHT-induced apoptosis, reducing Mcl-1 and XIAP levels. As we utilized NSCLC cell lines with K-Ras mutation, for which molecular target therapy is not available, and a *C. elegans* multivulva model with EGFR T790M-L858R representing TKI-resistant tumors, our data suggest that IHT and its combination with NR4A1 inhibition are potential strategies to treat NSCLCs.

## Supplementary Materials

The following are available online at https://www.mdpi.com/2218-273X/10/11/1521/s1. Figure S1: Isoharringtonine (IHT) inhibited the growth of NSCLC tumorspheroids independent of NR4A1; Figure S2: IHT induced mitochondria-mediated apoptosis in NCI-H460 independent of NR4A1; Figure S3: iNR4A1 significantly reduced the mRNA level of NR4A1; Figure S4: Low doses of IHT induced expression of p21 protein in NCI-H460 cells; Figure S5: IHT inhibited the viability of NSCLC tumorspheroids.
