# Supplementary material for: Isoharringtonine Induces Apoptosis of Non-Small Cell Lung Cancer Cells in Tumorspheroids via the Intrinsic Pathway
Source: Biomolecules. 2020 Nov 6;10(11):1521. doi: 10.3390/biom10111521 (PMC7694770; doi:10.3390/biom10111521)
Supplement: Supplementary file 1 [file biomolecules-10-01521-s001.pdf]

Supplementary Materials

Supplementary Figure 1. Isoharringtonine (IHT) inhibited the growth of NSCLC tumorspheroids independent of NR4A1.

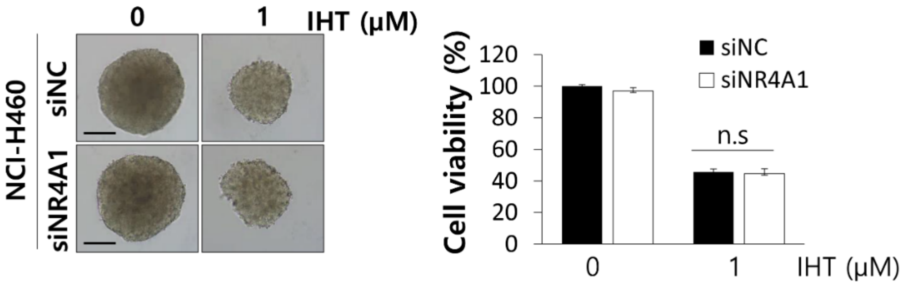

NCI-H460 tumorspheroids transfected with siNR4A1 were treated with 1 μM IHT for 48 h, and cell viability was measured. Data are presented as mean ± SD. n.s., not significant.

Supplementary Figure 2. IHT induced mitochondria-mediated apoptosis in NCI-H460 independent of NR4A1.

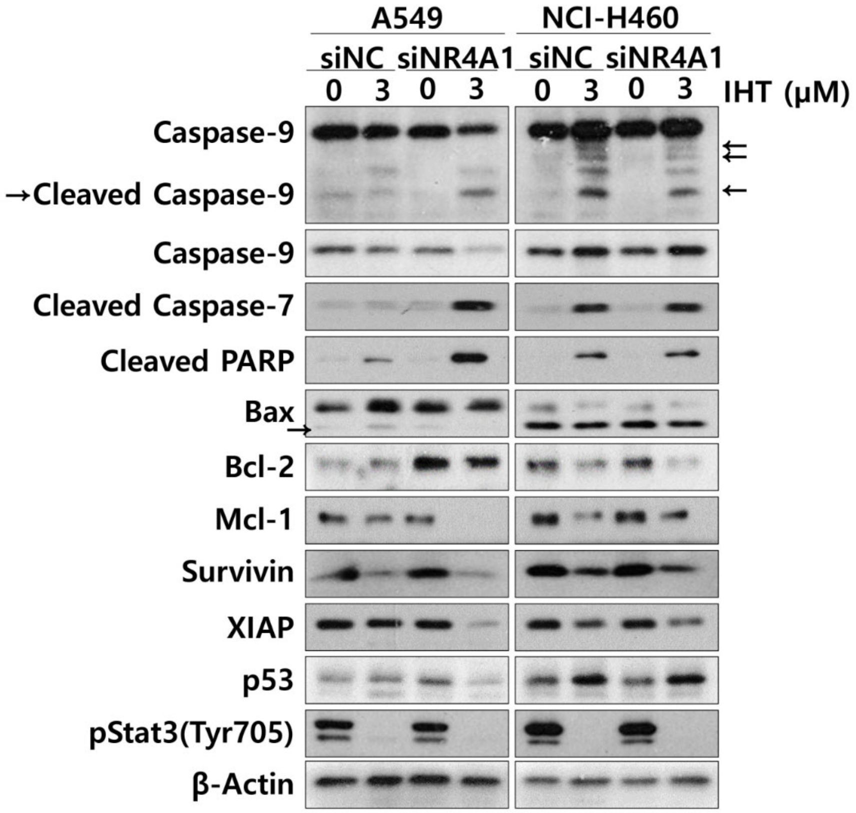

siRNA-transfected A549 and NCI-H460 tumorspheroids were treated with 3 μM IHT for 48 h, and the expression of apoptosis-related proteins was analyzed by western blotting. β-Actin was used as a loading control.

Supplementary Figure 3. iNR4A1 significantly reduced the mRNA level of NR4A1.

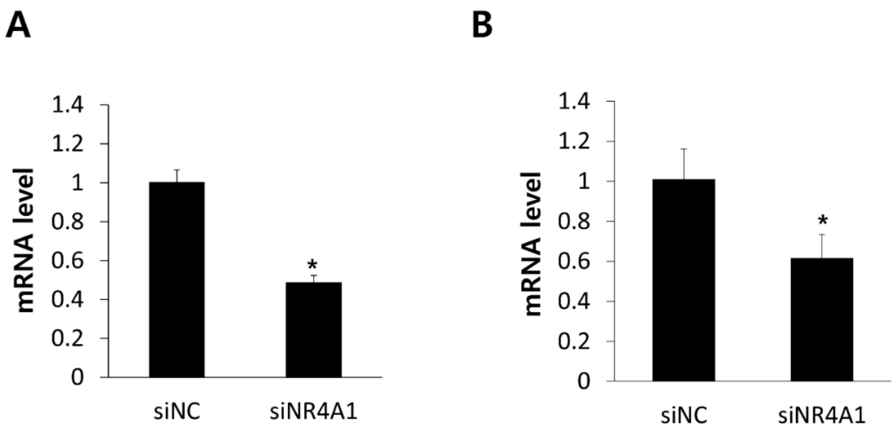

(A) A549 cells were transfected with siNR4A1 for 48h, and mRNA level of NR4A1 was analyzed by qPCR. (B) Xenograft tumor were transfected with siNR4A1 via electroporation for 48h, and mRNA level of NR4A1 was analyzed by qPCR. Data are presented as mean ± SD. *, significantly different from control ($p < 0.01$).

Supplementary Figure 4. Low doses of IHT induced expression of p21 protein in NCI-H460 cells.

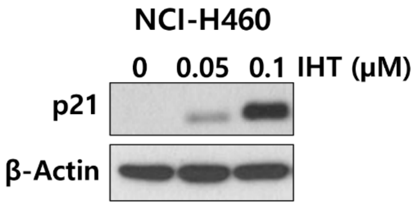

2D-cultured NCI-H460 cells were treated at the indicated concentrations of IHT for 48 h, and p21 expression was analyzed by western blotting.

Supplementary Figure 5. IHT inhibited the viability of NSCLC tumorspheroids.

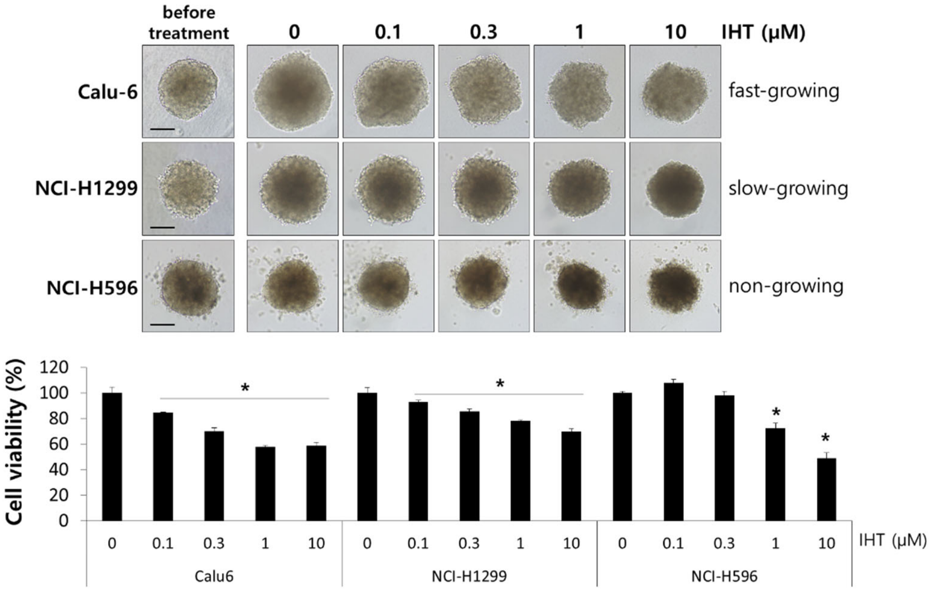

Two days after ULA plating, Calu-6, NCI-H1299, and NCI-H596 tumorspheroids were treated for 48h with the indicated concentrations of IHT. Cell viability was measured by the CellTiter-Glo 3D cell viability assay. Scale bars, 200 µm. *, significantly different from control ($p < 0.05$).
